# Iontophoresis-driven microneedle patch for the active transdermal delivery of vaccine macromolecules

**DOI:** 10.1038/s41378-023-00515-1

**Published:** 2023-03-27

**Authors:** Ying Zheng, Rui Ye, Xia Gong, Jingbo Yang, Bin Liu, Yunsheng Xu, Gang Nie, Xi Xie, Lelun Jiang

**Affiliations:** 1 Guangdong Provincial Key Laboratory of Sensor Technology and Biomedical Instrument, School of Biomedical Engineering, Shenzhen Campus of Sun Yat-Sen University, Shenzhen, 518107 PR China; 2 Department of Dermatovenereology, The Seventh Affiliated Hospital, Sun Yat-sen University, Shenzhen, 518107 PR China; 3 State Key Laboratory of Optoelectronic Materials and Technologies, School of Electronics and Information Technology, Sun Yat-sen University, Guangzhou, 510006 PR China

**Keywords:** Electrical and electronic engineering, Electronic properties and materials

## Abstract

COVID-19 has seriously threatened public health, and transdermal vaccination is an effective way to prevent pathogen infection. Microneedles (MNs) can damage the stratum corneum to allow passive diffusion of vaccine macromolecules, but the delivery efficiency is low, while iontophoresis can actively promote transdermal delivery but fails to transport vaccine macromolecules due to the barrier of the stratum corneum. Herein, we developed a wearable iontophoresis-driven MN patch and its iontophoresis-driven device for active and efficient transdermal vaccine macromolecule delivery. Polyacrylamide/chitosan hydrogels with good biocompatibility, excellent conductivity, high elasticity, and a large loading capacity were prepared as the key component for vaccine storage and active iontophoresis. The transdermal vaccine delivery strategy of the iontophoresis-driven MN patch is “press and poke, iontophoresis-driven delivery, and immune response”. We demonstrated that the synergistic effect of MN puncture and iontophoresis significantly promoted transdermal vaccine delivery efficiency. In vitro experiments showed that the amount of ovalbumin delivered transdermally using the iontophoresis-driven MN patch could be controlled by the iontophoresis current. In vivo immunization studies in BALB/c mice demonstrated that transdermal inoculation of ovalbumin using an iontophoresis-driven MN patch induced an effective immune response that was even stronger than that of traditional intramuscular injection. Moreover, there was little concern about the biosafety of the iontophoresis-driven MN patch. This delivery system has a low cost, is user-friendly, and displays active delivery, showing great potential for vaccine self-administration at home.

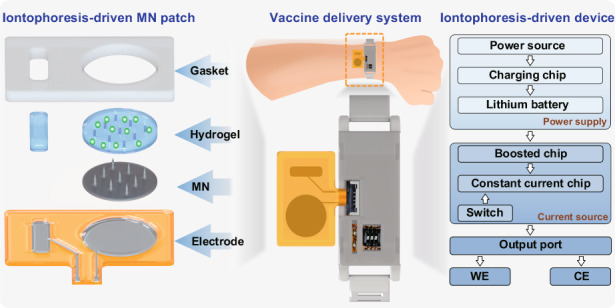

## Introduction

Coronavirus disease 2019 (COVID-19) has evolved into a pandemic and poses a great threat to public health^1,2^. Vaccination provides an effective way to prevent pathogen infection^3,4^. The development of effective vaccines is one key to preventing epidemic transmission and achieving herd immunity. To date, preventive vaccines have eliminated many diseases, such as smallpox and poliomyelitis^5^, and have the potential to curb the occurrence of many other common infectious diseases and halt the global spread of the emerging COVID-19 pandemic^6^. Most vaccines are given by intramuscular (IM) injection, but this method has many inherent limitations: fear of needles in children and adolescents^7^, procedural pain^8^, and infections caused by the repeated use of needles^9^. Due to the rich network of immune cells in the skin, transdermal immunization is becoming an attractive alternative^10^. However, owing to the stratum corneum (SC) barrier, only small molecule vaccines (~<500 Da) can be effectively administered through the skin and enter the systemic circulation, so macromolecular vaccines have poor skin permeability and low bioavailability^11–13^. To break through the barrier of the SC and improve the transdermal permeation efficiency of vaccines, transdermal vaccination strategies have been developed to enhance the transdermal immune effect.

Microneedles (MNs) provide a new solution in the field of transdermal vaccine delivery due to their unique advantages of painless minimally invasive delivery, self-administration, and improved permeability^14^. The length of the MN is specially designed to give it the ability to penetrate the SC without stimulating nerve endings. The epidermis and dermis have a dense network of immune cells, such as Langerhans cells (LCs) and dermal dendritic cells (DDCs), whose anatomical sites can be reached by the MN during puncture^15^. MNs produce microchannels through the SC by skin penetration, allowing the vaccine macromolecules to permeate the anatomical sites of the immune cells^16,17^, so transdermal immunity using MNs is promising. However, transdermal vaccine delivery using MNs usually relies on passive diffusion via poked microchannels, which severely limits the transport speed and efficiency into the skin^18^. On the other hand, the iontophoresis technique can actively drive ionized and hydrophilic small molecules through the SC layer using a mild current (usually <0.5 mA/cm^2^), and therefore, this strategy has been applied in transdermal vaccine delivery and dermatological treatment^19,20^. Iontophoresis can control the transport process of vaccine molecules via the applied electricity because of electromigration and electroosmosis^21–25^. Iontophoresis has been successful in promoting the transdermal delivery of small hydrophilic molecules, but it usually fails in the transdermal delivery of macromolecules (e.g., proteins with a molecular weight >13 kDa) due to the barrier of SC^26–28^. However, most vaccines, such as nucleic acid vaccines, are macromolecules, which are difficult to administer transdermally into the skin using iontophoresis. Therefore, it is promising to utilize the synergistic advantages of macromolecule delivery using MNs and active delivery using iontophoresis to overcome the limitations of the low delivery efficiency of MNs and the failure of macromolecule delivery of iontophoresis.

**Fig. 1:**
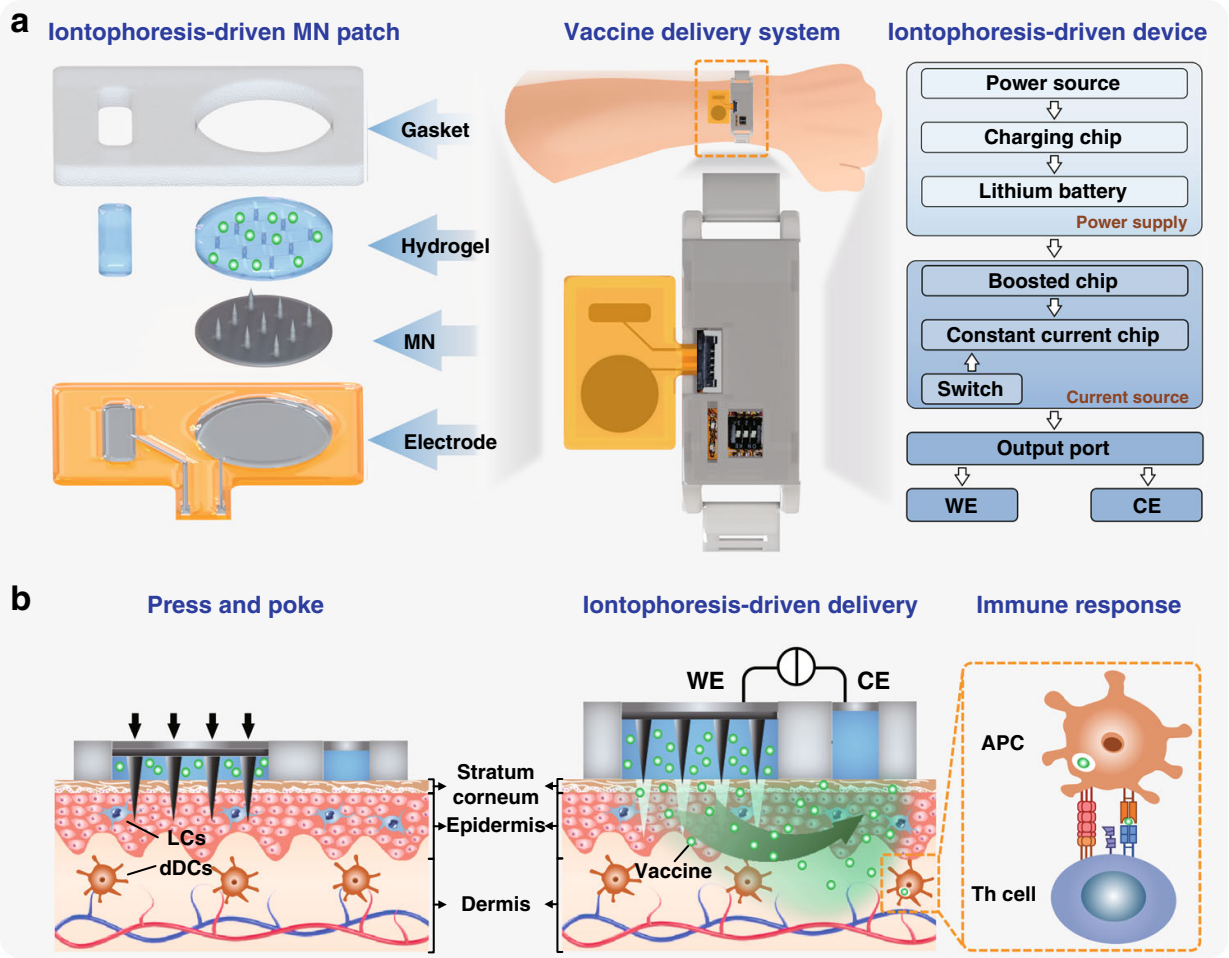
Schematic illustration of the wearable iontophoresis-driven MN delivery system. **a** Schematic illustration of the wearable delivery system, which mainly consists of the iontophoresis-driven MN patch and the iontophoresis-driven device. The iontophoresis-driven MN patch is composed of Ag/AgCl electrodes on a flexible PI, solid MNs, hydrogel blocks, and an adhesive impermeable gasket. The iontophoresis-driven device can supply power and output a constant current for iontophoresis-driven vaccine delivery. **b** The transdermal vaccine delivery strategy of the iontophoresis-driven MN patch is “press and poke, iontophoresis-driven delivery, and immune response”

In this work, we developed a wearable iontophoresis-driven MN system for the efficient and active transdermal delivery of macromolecular vaccines, as shown in Fig. 1 and Video S1. This system mainly consists of an iontophoresis-driven MN patch and an iontophoresis-driven device. The transdermal vaccine delivery strategy of the iontophoresis-driven MN patch is “press and poke, iontophoresis-driven delivery, and immune response” in which solid MNs are first pressed onto the skin to create microchannels through the SC and then automatically retracted. The vaccine macromolecules are then delivered through these created microchannels via passive diffusion and iontophoresis and captured by antigen-presenting cells in the epidermis and dermis, finally activating the cells to exert immune effects. The iontophoresis-driven MN patch combines the advantages of the MN and iontophoresis techniques and significantly promotes the transdermal delivery efficiency of vaccine macromolecules. Moreover, a flexible polyacrylamide/chitosan hydrogel with high loading ability and good electrical conductivity was selected as the vaccine storage chamber, which not only addressed the limitation of low vaccine loading of dissolvable MNs but also guaranteed stable conductivity between the electrodes and skin during iontophoresis. In vivo transdermal immunization demonstrated that the iontophoresis-driven MN patch could achieve an effective immune response that was even stronger than that using traditional intramuscular injection. Therefore, our iontophoresis-driven MN system is low-cost and user-friendly, showing promise as an alternative for vaccine self-administration at home.

## Results and discussion

### Transdermal vaccine delivery strategy of iontophoresis-driven MN patches

A wearable iontophoresis-driven MN delivery system composed of an iontophoresis-driven MN patch and an iontophoresis-driven device is proposed for the transdermal delivery of vaccine macromolecules, as shown in Fig. 1a and Video S1. The iontophoresis-driven MN patch mainly consists of Ag/AgCl electrodes on the flexible PI, solid MNs, hydrogel blocks, and a double-sided adhesive impermeable gasket. The circular hydrogel blocks are conductive and are used to load the vaccine antigen. The MN base is attached to the working electrode (WE), and the microneedles penetrate the circular hydrogel. The flexible electrodes, MNs, and hydrogel blocks are assembled in an impermeable gasket. The adhesive impermeable gasket can hold each component firmly in place, ensuring a stable iontophoresis current during vaccine delivery. Iontophoresis-driven MN patches are compressible due to the high elasticity of the hydrogel and gasket. In the iontophoresis-driven MN patches, the vaccine is loaded in the circular hydrogel, the skin is compressed and poked to create microchannels via the MNs, and the vaccine is delivered through the microchannels from the hydrogel via active iontophoresis and passive diffusion. The wearable iontophoresis-driven device can supply power and output a constant current for active iontophoresis-driven vaccine delivery.

The transdermal vaccine delivery strategy of the Iontophoresis-driven MN patch is “press and poke, iontophoresis-driven delivery, and immune response”, as shown in Fig. 1b and Video S1.

1. Press and poke. The iontophoresis-driven MN patch is conformably adhered to the skin. When the patch is pressed using a finger, the solid MN penetrates through the hydrogel and the SC, creating transient microchannels in the skin. Upon removal of the compression, the MN detaches from the skin and retracts into the hydrogel due to the elastic rebound energy of the gasket and hydrogels. Owing to the viscoelasticity and self-healing ability of the skin, the MN-induced microchannels will gradually close and heal upon retraction of the MNs. Moreover, the patch can be repeatedly pressed to reopen the microchannels in the skin and initiate another cycle of transdermal vaccine delivery.
2. Iontophoresis-driven vaccine delivery. The vaccine in the hydrogel passively diffuses into skin via the poked microchannels along the concentration gradient according to Fick’s diffusion law. The passive diffusion rate is mainly determined by the concentration of the vaccine in the hydrogel and created microchannels. A mild electric current is applied between the WE and counter electrode (CE) to initiate iontophoresis and actively drive the charged vaccine macromolecules to permeate into the skin through the poked microchannels under the main driving forces of electromigration and electroosmosis^29^. Most of the electroosmotic flow during iontophoresis migrates along the low-resistance and preferential pathways associated primarily with the microchannels^30–32^. The iontophoresis-driven delivery rate of the vaccine is mainly determined by the formulation characteristics of the vaccine, the microchannels poked by the MNs, and the iontophoresis current and duration^33^. Moreover, the combination of passive diffusion and active iontophoresis-driven delivery may lead to a synergistic enhancement in the transdermal delivery efficiency of vaccines.
3. Immune response. A dense network of immune cells is distributed in the epidermis and dermis of the skin. Upon delivery into the skin, the vaccine is captured by antigen-presenting cells (APCs), such as LCs and DDCs. After antigen stimulation, APCs migrate to the draining lymph nodes and activate Th lymphocytes to play a role in immunity^34^. APCs located in the epidermis and dermis activate the cells to exert an immune effect. Therefore, the agent stimulates the body’s immune system to recognize the agent as a threat, destroy it, and further recognize and destroy any of the microorganisms associated with that agent that it may encounter in the future.

### Fabrication and characterization of the wearable iontophoresis-driven MN delivery system

**Fig. 2:**
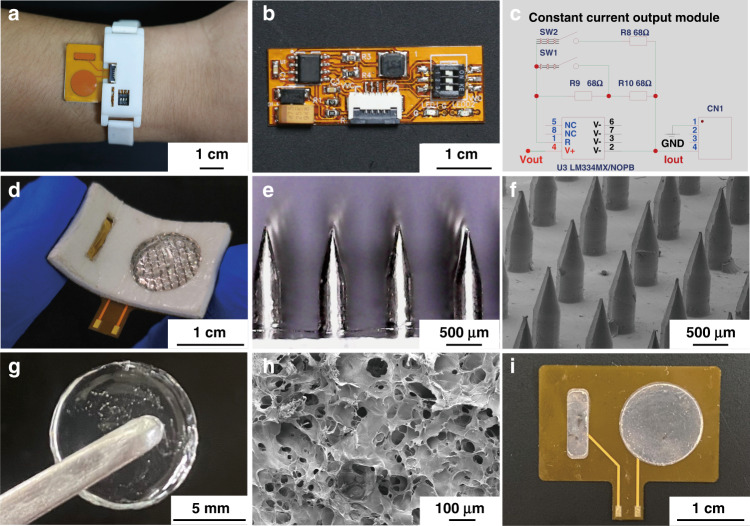
Characterization of the iontophoresis-driven MN delivery system. **a** Image of the iontophoresis-driven MN delivery system worn on a wrist. **b** The flexible PCB circuit of the iontophoresis-driven device. **c** Schematic diagram of the constant current output module. LM334 is the core chip of the constant current output circuit. **d** Image of a flexible iontophoresis-driven MN patch. **e** Optical image of the solid MNs. **f** SEM image of the solid MNs. **g** Optical image of the circular hydrogel block loaded with the vaccine. **h** SEM image of the freeze-dried hydrogel. **i** Optical image of the Ag/AgCl electrodes on the flexible PI

A wearable iontophoresis-driven MN delivery system was developed, as shown in Fig. 2a. It consists of the iontophoresis-driven MN patch and the iontophoresis-driven device. The iontophoresis-driven MN patch is adhered to the wrist with a size of 28 × 18 × 2 mm^3^. The iontophoresis-driven device is encapsulated in a white 3D-printed insulating shell with a size of 53 × 19 × 10 mm^3^, which can be worn on the wrist using a custom watchband. The iontophoresis-driven device outputs the iontophoresis current to the patch using a flexible PCB connector. The wearable iontophoresis-driven MN delivery system is small and light weight (only 18 g), enabling self-administration in daily life.

The PCB diagram and detailed circuit principle of the iontophoresis-driven device are shown in Fig. 2b, c and Figs. S1–7, respectively. The iontophoresis-driven circuit is mainly composed of the charging module, the boosted module, and the constant current output module, as shown in Fig. S2. The iontophoresis-driven circuit is powered by a lithium battery that can be recharged via a micro-USB port. The boosted module adjusts the output voltage of the lithium battery at the designed voltage for the constant current chip. The constant current output module provides a constant iontophoresis-driven current for vaccine delivery. LM334 is the core chip of the constant current output circuit, as shown in Fig. 2c. The charging and constant current output performance of the iontophoresis-driven device were verified (Fig. S8). The output voltage of the iontophoresis-driven device is linearly proportional to the load resistance, indicating that the current output is independent of the load resistance from 5 to 20 kΩ (typical skin impedance is ~10 kΩ). Moreover, the iontophoresis-driven device can be stably maintained at 0.5 mA for 1 h with little fluctuation.

A flexible iontophoresis-driven MN patch was assembled with Ag/AgCl electrodes on the flexible PI, solid MNs, hydrogel blocks, and a gasket, as shown in Fig. 2d. The impermeable gasket can conform and tightly stick to the curved skin surface, thereby avoiding interstitial fluid leakage and preventing hazardous infection. Figure 2e, f shows the optical and SEM images of the solid MNs fabricated by micromachining from stainless steel 316 L, which has high mechanical strength and biocompatibility for skin penetration^35^. Sixty-nine microneedles with conical tips and cylindrical bodies are uniformly arranged on the Φ12.4 mm cylindrical substrate. The average height, tip radius, and base diameter of the MNs are ~800, 20, and 400 µm, respectively. The adjacent microneedle distance is ~1200 µm. The solid MNs possess sharp tips for effective skin penetration. Figure 2g shows a cylindrical hydrogel block as the vaccine reservoir made of polyacrylamide (PAM)/chitosan for vaccine loading. The hydrogel block is compressible and conductive, can come in close contact with the curved skin and maintains good conductivity between the skin and the electrode. Figure 2h presents the morphology of the freeze-dried hydrogel. The hydrogel is porous, in which the vaccine and water are captured and stored. Figure 2i presents a pair of iontophoresis Ag/AgCl electrodes on a flexible PI. The Ag/AgCl electrodes were prepared by sputter coating Ag film and chlorination of Ag^36^.

### Basic performance of the iontophoresis-driven MN patch

**Fig. 3:**
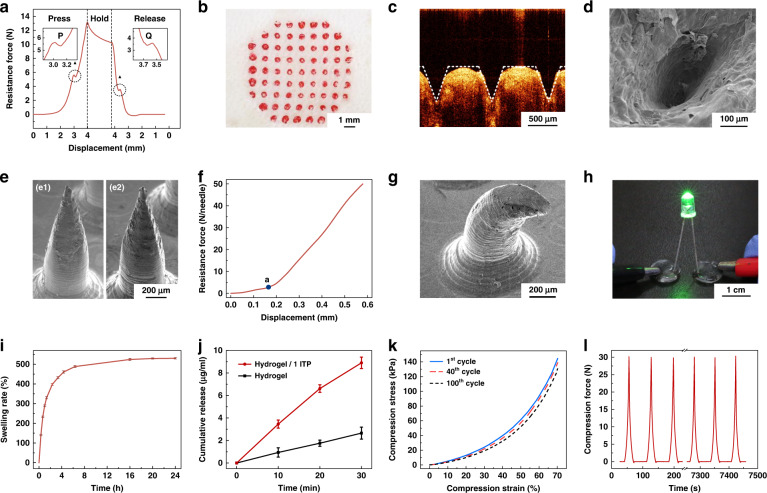
Basic performance of the iontophoresis-driven MN patch. **a** The relationship between the resistance force and loading displacement during the “press and poke” stage of MN patch application. The penetration force per microneedle was ~81.2 mN. **b** Optical image of rat skin poked by the MN patch. The MN-poked microchannels are marked with red dye. **c** OCT image of rat skin poked by the MN patch, demonstrating successful skin penetration. **d** SEM image of a microchannel in rat skin poked by the MN patch. **e** SEM image of a magnified microneedle after (e1) the 25th cycle and (e2) the 50th cycle of skin penetration. **f** The relationship between the resistance force and loading displacement during the MN patch fracture test. **g** SEM image of the microneedle after the fracture test. The microneedle was bent but not broken. **h** The hydrogel was connected to a circuit, and a green LED became lit. **i** Swelling rate of the hydrogel. The swelling rate reached ~530% in 24 h (data are mean ± SD, *n* = 3). **j** The cumulative release of OVA from the hydrogel in PBS with/without application of a 1 mA/cm^2^ iontophoresis current (data are mean ± SD, *n* = 3). **k** The compression stress−strain curves of the hydrogel for the 1st, 40th, and 100th cycles. **l** The cyclic compression force curves of the vaccine-loaded hydrogel block with a maximum strain of 70%

Since the iontophoresis-driven MN patch promotes vaccine delivery through poked microchannels, the skin penetration performance of the MN patch was investigated, as shown in Fig. 3a. During the “press and poke” stage, the resistance force increased with loading displacement until the maximum stress of the skin at the microneedle tips exceeded the rupture limit of skin, causing a sudden drop in force at point “P”. The critical penetration force of the MN patch was ~5.6 N, which is lower than typical thumb pressure^37^, indicating easy skin penetration to create microchannels by compression of the iontophoresis-driven MN patch. It was further demonstrated that neatly arranged microchannels formed in the poked skin (Fig. 3b). The distribution of the poked microchannels was consistent with that of MNs, demonstrating a high skin penetration rate (almost 100%). The depth and base diameter of the poked microchannels were ~500 and 250 μm, respectively, as shown in Fig. 3c, d. This result indicated that the MN patch could penetrate through the stratum corneum layer (~10–20 μm^38^) to promote transdermal vaccine delivery. Upon removal of the compression of the MN patch, the resistance force decreased, which was mainly determined by the elastic recovery of the skin and MN patch and the friction between the MN and skin. Once the MNs were detached from the skin, the friction force became zero, causing a force increase at point “Q” (~3.5 N)^39^. Moreover, the above “press and release” actions on rat skin using the MN patch were repeated for 50 cycles, and the morphology of MNs varied little without damage (Fig. 3e), indicating that the MN patch can repeatedly poke the skin. The fracture performance of the MN patch was further investigated, as shown in Fig. 3 f. The resistance force increased with loading displacement. The MN tips were bent at almost 90° without breakage at point “a” (2.6 N/needle)^40^, as shown in Fig. 3g. The fracture force of the MN patch was much higher than the skin penetration force (0.08 N/needle) due to the high mechanical strength of the solid stainless steel MNs, indicating that these MN patches can penetrate the skin without bending. Therefore, the MN patches can easily penetrate skin, produce microchannels, and retract from skin without any damage, avoiding MN breakage in the skin, foreign body sensation, and the possibility of inflammation.

The hydrogel was connected with a circuit, and a green light-emitting diode became lit (Fig. 3h), demonstrating good conductivity. The conductivity of the hydrogel was 0.16 S/m measured by the two-point probe method. The hydrogel, as a conductive medium, effectively guarantees conductivity between the electrode and skin. Ovalbumin (OVA, a model antigen) was loaded into the hydrogel via its swelling behavior. The weight of the hydrogel rapidly increased in the first 4 h and reached swelling equilibrium in ~8 h. The final swelling rate was 530.2 ± 3.1% in 24 h (Fig. 3i). The OVA loading performance of the hydrogel was investigated. The maximum OVA loading capacity of the hydrogel (40 mg) reached 151.0 ± 16.5 μg with a loading efficiency of ~60% when the hydrogel was soaked in 250 µL of OVA solution (1 mg/mL). Moreover, OVA-FITC was uniformly distributed in the hydrogel (Fig. S9). The OVA vaccine release performance of the hydrogel was examined, and the schematic diagram of the test is shown in Fig. S10. Figure 3j shows the cumulative release of OVA from the hydrogel with/without application of a 1 mA/cm^2^ iontophoresis current. The OVA release rates with and without the application of a 1 mA/cm^2^ iontophoresis current for 30 min were 8.90 ± 0.51 μg/mL and 2.66 ± 0.53 μg/mL, respectively. The current increased 3.3-fold upon application of the iontophoresis current compared with only passive diffusion. Moreover, the electrostatic adsorption of cationic chitosan on the negatively charged proteins is also a possible reason for the low passive diffusion rate of the hydrogel^41^. Figure 3k, l presents the cyclic compression stress‒strain and force‒time curves of the hydrogels with a maximum strain of 70%, respectively. The elasticity of the hydrogels varied little after 100 compression cycles. The compression stress reached ~130 kPa at 70% strain, and the compression force of the vaccine-loaded hydrogel block was ~30 N at 70% strain. As shown in Fig. S11, the shape of the hydrogel block displayed little variation, and no obvious damage was observed after 100 cycles of repeated compressions. Therefore, the hydrogel can be compressed with good elasticity for skin penetration and MN detachment.

### In vitro transdermal vaccine delivery performance

**Fig. 4:**
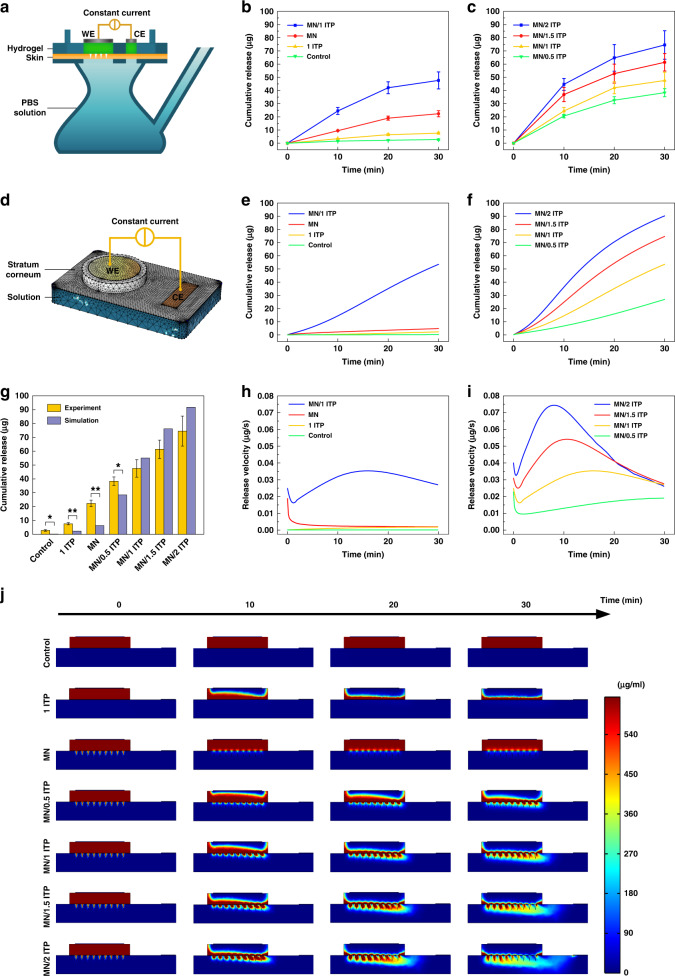
In vitro and numerically calculated transdermal OVA delivery performance of the iontophoresis-driven MN patches. **a** Schematic illustration of in vitro transdermal OVA delivery using the iontophoresis-driven MN patch assembled on a Franz diffusion cell. **b** In vitro cumulative amount of permeated OVA in various groups, including the control, 1 ITP, MN, and MN/1 ITP groups. **c** The effect of iontophoresis-driven current on in vitro transdermal OVA delivery (data are the mean ± SD, *n* = 3). **d** Schematic illustration of the FEA model for transdermal OVA delivery using an iontophoresis-driven MN patch. **e** The calculated cumulative amount of permeated OVA in the control, 1 ITP, MN, and MN/1 ITP groups. **f** The effect of iontophoresis-driven current on the calculated cumulative amount of permeated OVA in the MN/0.5 ITP, MN/1 ITP, MN/1.5 ITP, and MN/2 ITP groups. **g** Comparison of the final cumulative amount of permeated OVA between the in vitro experimental and calculated groups (data are mean ± SD, *n* = 3). **h** The calculated transdermal delivery speed of OVA in the control, 1 ITP, MN, and MN/1 ITP groups. **i** The effect of iontophoresis-driven current on the calculated transdermal OVA delivery speed in the MN/0.5 ITP, MN/1 ITP, MN/1.5 ITP, and MN/2 ITP groups. **j** The calculated transdermal OVA delivery process in the control, 1 ITP, MN, MN/0.5 ITP, MN/1 ITP, MN/1.5 ITP, and MN/2 ITP groups

To explore the transdermal vaccine delivery performance of the iontophoresis-driven MN patch, vertical Franz diffusion cells were designed in-house for in vitro permeation tests, as shown in Fig. 4a. The cathode iontophoresis method was employed in this work since OVA in PBS (pH 7.4) carries negative charges due to its isoelectric point of 4.75^42^. The effects of skin penetration by the MNs and iontophoresis on the in vitro OVA delivery were systematically investigated, as shown in Fig. 4b. The cumulative concentration of OVA increased with experimental time, and a significant difference in cumulative permeation was clearly observed among these groups after 30 min of administration. The cumulative amounts permeated in the control, MN, 1 ITP, and MN/1 ITP groups were 2.80 ± 0.59 μg, 22.35 ± 2.32 μg, 7.61 ± 0.78 μg, and 47.57 ± 6.31 μg, respectively. Under free diffusion, OVA gradually passed through intact skin and was significantly facilitated (almost 8-fold) via the created microchannels. The microchannels created by the MNs effectively overcame the skin barrier and were beneficial for increasing the permeation rate. Conventional iontophoresis could also promote OVA delivery (2.7-fold) compared with free diffusion through intact skin. Transdermal OVA delivery could be further be enhanced via the combination of skin penetration and iontophoresis. The cumulative OVA permeation amount in the MN/1 ITP group was ~17-fold that in the control group. The microchannels created by the MNs provide transdermal delivery routes for iontophoresis, and the MNs and iontophoresis can cooperate to further improve transdermal permeation.

The effect of the iontophoresis-driven current on the permeation rate was further investigated, as shown in Fig. 4c. The cumulative amounts permeated in the MN/0.5 ITP, MN/1 ITP, MN/1.5 ITP, and MN/2 ITP groups were 38.35 ± 3.04 μg, 47.57 ± 6.31 μg, 61.39 ± 6.61 μg, and 74.53 ± 10.82 μg, respectively. The cumulative amount of permeated OVA increased almost linearly with the iontophoresis-driven current (Fig. S12a). This significant increase is attributed to the positive effects of electromigration. The cumulative amount permeated in the MN/2 ITP group was the highest, which was approximately twofold that in the MN/0.5 ITP group. The linear relationship between the iontophoresis current and the permeation amount indicates that the amount of vaccine permeated through the skin can be controlled by the iontophoresis current.

### Numerical analysis of transdermal vaccine delivery

The synergistic permeation mechanism of skin penetration by the MNs and iontophoresis was further analyzed using the FEA method. The FEA model of the iontophoresis-driven MN patch for transdermal OVA delivery was established using COMSOL Multiphysics, as shown in Fig. 4d and Fig. S13. This model was analyzed using the potential coupling of Transport of Diluted Species and Electric Current Physics. The model parameters were set based on the in vitro transdermal delivery of OVA. Figure 4j and Video S2 show the concentration distribution during the permeation process for each simulation group. The OVA flow distribution of the MN and MN/ITP groups can be clearly observed. In particular, the OVA concentration in the MN/ITP groups was distributed along the electric field, demonstrating the transport function of iontophoresis. Based on the simulation results, the cumulative amount of permeated material and permeation rate were further calculated, as shown in Fig. 4e–i. The cumulative amounts of permeated material in the control, 1 ITP, MN, and MN/1 ITP groups were 0.17, 2.28, 6.32, and 55.08 μg, respectively, as shown in Fig. 4e. The MN/1 ITP group combined the MN and iontophoresis technologies and showed the highest permeability. Moreover, the cumulative amount permeated in the MN/ITP group also increased with the application of iontophoresis current (Fig. 4f and Fig. S12b), which is consistent with the in vitro experimental results, demonstrating the effectiveness and controllability of active iontophoresis on vaccine delivery.

According to Fig. 4e, the permeation rate in the above groups could be further calculated, as shown in Fig. 4h. The permeation rates in the control and 1 ITP groups were low due to the ideal skin barrier of the stratum corneum without skin appendages (such as hair follicles) in the simulation. The permeation rate in the MN group rapidly decreased and then reached a balance under passive diffusion via the created microchannels. According to Fick’s diffusion law, the diffusion rate is proportional to the concentration gradient, and the concentration gradient gradually decreased between the MN patch and receptor chamber. The permeation rate in the MN/1 ITP group first decreased at the initial stage because passive diffusion plays the main role during OVA delivery at this point, then gradually increased because of the greater ability of iontophoresis to drive the vaccine via the microchannels created by the MNs, and finally decreased owing to the significant drop in the amount of vaccine stored in the patch. The MN/1 ITP group showed the highest permeation rate due to the combined effect of the MNs and iontophoresis compared with the control, MN, and 1 ITP groups. The permeation rate in the MN/ITP groups increased with iontophoresis current, as shown in Fig. 4i. The simulation and experimental permeation curves exhibited very similar tendencies, as shown in Fig. 4e, f and Fig. 4b, c. Therefore, we compared the calculated and in vitro experimental cumulative amount of permeated OVA (Fig. 4g), and the MN/ITP groups showed a very similar tendency, further demonstrating that vaccine delivery via iontophoresis-driven MN patches can be controlled by the iontophoresis current.

### Immunization performance

**Fig. 5:**
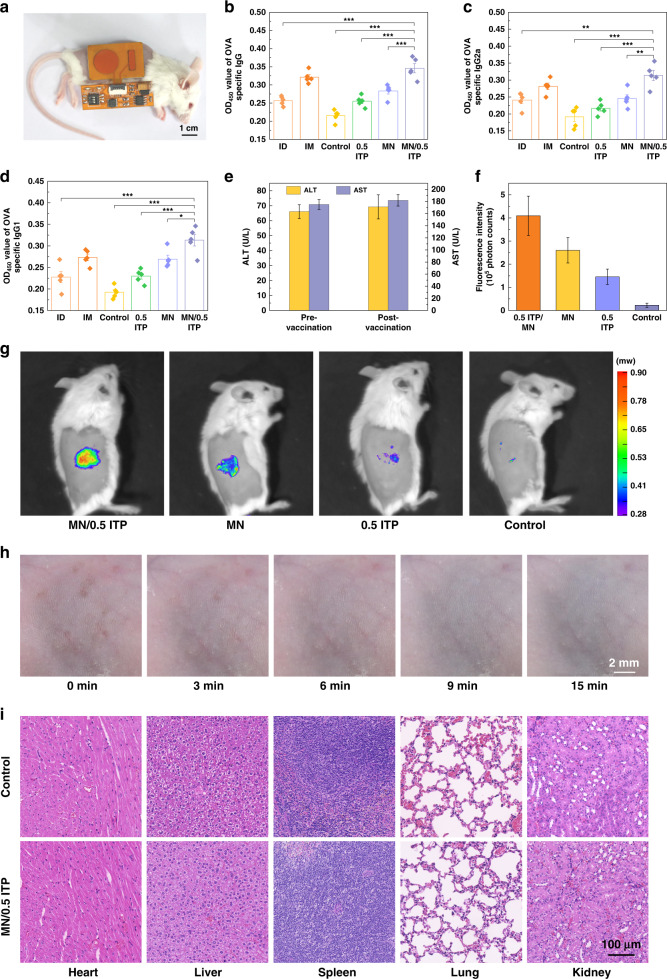
In vivo immunization studies of the iontophoresis-driven MN patch and its iontophoresis-driven device. **a** Transdermal OVA delivery for the vaccination of BALB/c mice using an iontophoresis-driven MN patch and its device. Iontophoresis-driven MN patches loaded with OVA were bound to the right abdominal skin of mice with shaved hair. The OVA-specific **b** IgG, **c** IgG1, and **d** IgG2a levels in the serum of the control, intradermal injection, intramuscular injection, MN, 0.5 ITP, and MN/0.5 ITP groups (data are mean± SD, *n* = 5 per group). **e** ALT and AST levels in the serum of MN/0.5 ITP group mice pre- and postvaccination (data are mean ± SD, *n* = 3). **f**, **g** IVIS images and the corresponding fluorescent area in the control, MN, 0.5 ITP, and MN/0.5 ITP groups (data are mean ± SD, *n* = 3). **h** Mouse skin recovery after vaccination with the iontophoresis-driven MN patch (MN puncture and 0.5 mA/cm^2^ iontophoresis for 30 min). The treated skin self-recovered within 15 min. **i** Hematoxylin and eosin (H&E) staining of the main organs (heart, liver, spleen, lung and kidney) of the mice in the MN/0.5 ITP and control groups

The levels of antigen-specific IgG and its subtypes (IgG1 and IgG2a) are positively correlated with the intensity of the immune response^43^. IgG1 and IgG2a antibody levels well reflect the intensity of Th2 and Th1 responses, respectively^44^. To study the antibody response induced by vaccines administered by the iontophoresis-driven MN delivery system, OVA was selected as the model antigen to induce the antibody response in BALB/c mice (Fig. 5a). The intensity of the immune response of the mice in the different groups (including intradermal injection, intramuscular injection, control, MN, 0.5 ITP, and MN/0.5 ITP group) was tested. The control group received transdermal vaccination using an iontophoresis-driven MN patch without MN poke and iontophoresis. The levels of OVA-specific IgG, IgG1, and IgG2a in mouse serum were measured two weeks after booster vaccination (Fig. 5b–d). As shown in Fig. 5b–d, all antibody levels of OVA-specific IgG, IgG1, and IgG2a in the MN/0.5 ITP group were slightly higher than those in the intramuscular injection group, indicating that transdermal vaccination with MN/0.5 ITP could produce similar or even higher immune response than typical intramuscular injection. However, typical intramuscular injection is slightly painful and requires professional operation by medical staff. The antibody levels of OVA-specific IgG, IgG1, and IgG2a in the MN/0.5 ITP group were significantly higher than those in the intradermal injection, control, MN and 0.5 ITP groups, indicating that the combination of the MN and iontophoresis techniques not only promoted transdermal vaccine delivery but also contributed to promoting both Th1- and Th2-type humoral immune responses. The antibody levels of OVA-specific IgG, IgG1, and IgG2a in the MN group were superior to those in the 0.5 ITP group due to the limited transdermal permeability of the macromolecule OVA (~43 kDa) driven by iontophoresis through intact skin. MNs can destroy the barrier layer of the stratum corneum to produce microchannels for passive diffusion and active iontophoresis. Above all, the synergistic effect of skin penetration and iontophoresis can enhance transdermal vaccine delivery. Elevated ALT and AST levels are associated with hepatocyte injury^45^. Transdermal vaccine delivery using an iontophoresis-driven MN patch was performed in mice for 30 min, serum was collected 24 h later, and the ALT and AST levels were analyzed, as shown in Fig. 5e. The ALT and AST levels of mice were within the normal range pre- and postvaccination without significant differences, indicating good biocompatibility of the iontophoresis-driven MN patch.

In vivo transdermal OVA delivery via the iontophoresis-driven MN patch was further studied using a small animal in vivo imaging system (IVIS). The near-infrared (NIR) wavelength range is favorable for in vivo imaging^46^, so Cy7-labeled OVA (Cy7-OVA) was selected and loaded into hydrogels for transdermal delivery. The in vivo fluorescence images of four groups (control, MN, 0.5 ITP, and MN/0.5 ITP group) after 30 min of treatment were observed, as shown in Fig. 5f, g. The fluorescence intensity in the MN/0.5 ITP group was the strongest, and its fluorescence area was the largest, suggesting that the combination of the MN and iontophoresis techniques could effectively deliver macromolecular OVA into the skin. Both the MN and 0.5 ITP groups promoted OVA transport into the skin. However, the control group without skin penetration or iontophoresis showed extremely low fluorescence intensity, demonstrating that transdermal delivery of the macromolecule OVA by passive diffusion was inefficient.

Skin penetration using MNs and iontophoresis may cause some skin damage and irritation^24,47^, so the ability of the skin to recover after treatment with iontophoresis-driven MN patches was evaluated. As shown in Fig. S14, the micropores in the mouse back skins that had been poked by the MNs healed within 40 min without erythema or lesions. Moreover, skin treated with MN puncture and 30 min of 0.5 mA/cm^2^ iontophoresis was examined, as shown in Fig. 5h. Upon iontophoresis, erythema could be observed on the skin poked by MNs, which might be attributed to the decrease in skin tolerance after MN puncture. However, these mild symptoms completely resolved within 15 min; thus, treatment combining MN puncture with iontophoresis is relatively safe. The biosafety of the iontophoresis-driven MN patch in terms of the skin, heart, liver, spleen, lungs and kidneys of the mice after patch administration was further investigated, as shown in Fig. 5i and Fig. S15. Histopathological examination (H&E staining) was conducted. Compared with the control group, pathological sections of the mouse organs in the MN/0.5 ITP group showed that the tissues of each organ were normal, including intact myocardial fiber tissue, normal liver parenchyma cells, dense rounding of the renal corpuscles, filling of the lungs with alveoli, and no obvious inflammatory cell infiltration in the skin. Therefore, transdermal vaccine delivery caused no significant pathological changes or toxicity to visceral tissues. These experimental results demonstrate that iontophoresis-driven MN patches provide a safe platform for transdermal vaccine delivery.

## Conclusion

We developed an iontophoresis-driven MN patch and a portable iontophoresis-driven device for efficient and controllable transdermal immunization. The iontophoresis-driven MN patch integrated the technologies of MNs and iontophoresis well, and the transdermal vaccine delivery strategy was “press and poke, iontophoresis-driven delivery, and immune response”. Hydrogel blocks with good biocompatibility, excellent conductivity, high elasticity, and a large loading capacity were prepared as the key component of the MN patches for vaccine storage and active iontophoresis. In vitro experiments demonstrated that iontophoresis-driven MN patches could control the transdermal OVA delivery process by tuning the iontophoresis current. Moreover, both in vitro and in vivo experiments demonstrated that the synergistic effect of skin penetration using MNs and iontophoresis could enhance the transdermal delivery efficiency of vaccine macromolecules. Iontophoresis-driven MN patches applied with a mild iontophoresis current raised few safety concerns. Above all, the wearable iontophoresis-driven MN system combining MNs with iontophoresis offers a promising strategy for achieving transdermal immunity in a painless and actively controlled manner.

## Experimental

### Ethics statement

All animal procedures conducted in this work were reviewed, approved, and supervised by the Institutional Animal Care and Use Committee at Sun Yat-Sen University (Approval Number: IACUC–DD–16–0904).

### Materials and animals

Chitosan, acrylamide, ammonium persulfate and N,N′-methylene bisacrylamide (Macklin, China) were purchased for the fabrication of the hydrogel. Ovalbumin (OVA) was purchased from Sigma, USA. OVA-FITC was purchased from Solarbio (China). OVA-Cy7 (Qiyuebio, China) was purchased for in vivo imaging of the mice.

Sprague–Dawley rats (male, 200 ± 30 g) were provided by the Xinhua Experimental Animal Farm (Guangzhou, China). Fresh rat skin was prepared by removal of hair and subcutaneous fat for mechanical tests and in vitro transdermal vaccine permeation tests. BALB/c mice (female, 18 ± 2 g, 6-week old) were purchased for transdermal immunity from Beijing Vital River Laboratory Animal Technology Co., Ltd.

### Fabrication and characterization of iontophoresis-driven MN patches

Iontophoresis-driven MN patches were assembled with Ag/AgCl electrodes on a flexible polyimide (PI), MNs, a conductive hydrogel and medical impermeable double-sided adhesive gasket, as shown in Fig. 1a. The Ag/AgCl electrode was prepared by chlorination of the Ag electrode with FeCl_3_ solution (0.1 M)^36^. Solid MNs were fabricated by micromilling 316 L stainless steel, which has been widely applied in medical implants, as it shows good mechanical strength and biocompatibility^48^. Polyacrylamide/chitosan vaccine-loaded hydrogels were prepared by UV polymerization and replica molding^49,50^. The morphology of the MNs was observed using scanning electron microscopy (SEM, Quanta 400F, Oxford, Holland). The freeze-dried hydrogel was sprayed with gold, and the cross-sectional morphology was observed by SEM. The conductivity of the hydrogel was tested using the two-point probe method^50^. The swelling rate of the hydrogel was measured by soaking the hydrogel in sufficient PBS until the hydrogels reached swelling equilibrium at room temperature.

### Fabrication of the iontophoresis-driven device

A portable iontophoresis-driven device was developed to output a constant current for the iontophoresis-driven delivery of vaccines. A miniature printed circuit board (PCB) (size: 36.5 × 13.2 × 1.0 mm^3^) with ultralow power consumption for iontophoresis was designed using Altium Designer (Altium, Australia). The schematic circuit of the iontophoresis-driven device is shown in Fig. S1. The circuit was mainly composed of a charging module, a boosted module and a constant current output module, as shown in Figs. S2–7. The performance tests of the iontophoresis-driven device are shown in Fig. S8. The output current of the iontophoresis-driven device (0.5, 1, 1.5, and 2 mA) can be adjusted with a switch. Moreover, the insulating shell of the device was fabricated by a 3D printer (Sindon, 3DWOX, Korea) to encapsulate the PCB of the iontophoresis-driven circuit.

### Mechanical tests of the iontophoresis-driven MN patches

1. The skin penetration performance of the iontophoresis-driven MN patch was investigated using a universal material testing machine (Instron, 5543 A, Boston, USA), as shown in Fig. S16a. Rat skin was fixed on polystyrene foam. The iontophoresis-driven MN patch was gradually pressed to 4 mm, held for 10 s, and subsequently released. The press and release speed were 0.1 mm/s. The penetrated rat skin was soaked in tissue fixative solution (4% paraformaldehyde), and the skin holes were observed by optical coherence tomography (OCT, HSO-2000, TEK SQRAY, China) and SEM. The mechanical stability of the MNs was studied by repeating the press-release actions at different locations on the rat skin for 50 cycles. The morphology of MNs after 25 and 50 cycles was observed by SEM.
2. The fracture performance of MNs was tested using a universal material testing machine (Instron, 5967, Boston, USA), as shown in Fig. S16b. The MNs were moved toward and pressed on a stainless steel plate at a speed of 0.1 mm/s until the resistance force reached 3.5 kN. The pressed MNs were observed using SEM and an ultra-deep field microscope (Keyence, VHX-5000, Osaka, Japan), as shown in Fig. S17.
3. The mechanical properties of the hydrogel were tested using a universal material testing machine. The cylindrical hydrogel block was compressed at a speed of 0.1 mm/s. The hydrogel block was repeatedly compressed for 100 cycles at a limit strain of 70%.

### In vitro OVA loading and delivery tests

The dried hydrogel (40 mg) was immersed in 250 µL of OVA solution (1 mg/mL) and placed at 4 °C for 48 h. The loading efficiency of the hydrogel was defined as the ratio of the actual amount of drug loaded to the total amount of drug applied. The in vitro OVA transdermal permeation efficiency of the iontophoresis-driven MN patch was tested using an intelligent transdermal testing instrument (TP-3A, Albert Tech., China). OVA-FITC was loaded into the hydrogel, and the distribution of OVA-FITC in the hydrogel was observed using confocal laser scanning microscopy (CLSM, FV3000, Olympus, Japan). The in vitro OVA delivery performance of the iontophoresis-driven MN patch was tested using a self-designed Franz diffusion cell, as shown in Figs. S18, 19. The receptor chamber (17 mL) was filled with phosphate-buffered saline (PBS, pH = 7.4). Rat skin samples with a size of 3 × 4 cm^2^ were fixed on the receptor chamber. The iontophoresis-driven MN patch was assembled on the donor chamber, and the iontophoresis-driven device was used to promote permeation. In vitro transdermal delivery tests were divided into 7 groups (*n* = 3), as listed in Table 1. (1) Control group: intact skin; (2) 1 ITP group: intact skin and iontophoresis with a current density of 1 mA/cm^2^; (3) MN group: skin penetration using MNs; (4) MN/0.5 ITP group: skin penetration using MNs and iontophoresis with a current density of 0.5 mA/cm^2^; (5) MN/1 ITP group: skin penetration using MNs and iontophoresis with a current density of 1 mA/cm^2^; (6) MN/1.5 ITP group: skin penetration using MNs and iontophoresis with a current density of 1.5 mA/cm^2^; and (7) MN/2 ITP group: skin penetration using MNs and iontophoresis with a current density of 2 mA/cm^2^. The OVA concentration in the diffusion cell was measured using a micro-BCA protein quantification kit (Leagene, pt0006-500t, Beijing, China).

**Table 1:** In vitro OVA transdermal delivery in the 7 groups

Group name	Skin treatment	Iontophoresis
Control group	Intact skin	0 mA/cm^2^
1 ITP group	Intact skin	1 mA/cm^2^
MN group	Skin penetration	0 mA/cm^2^
MN/0.5 ITP group	Skin penetration	0.5 mA/cm^2^
MN/1 ITP group	Skin penetration	1 mA/cm^2^
MN/1.5 ITP group	Skin penetration	1.5 mA/cm^2^
MN/2 ITP group	Skin penetration	2 mA/cm^2^

### Numerical simulations of transdermal vaccine delivery

The transdermal vaccine delivery process by the iontophoresis-driven MN patch combining skin penetration using MNs and iontophoresis was analyzed by the finite element analysis (FEA) method. The FEA model of the iontophoresis-driven MN patch was established using COMSOL Multiphysics, as shown in Fig. S13. The FEA models and parameters are listed in Table S1. The synergistic permeation effect of skin penetration using MNs and iontophoresis with the iontophoresis-driven MN patches was calculated using potential coupling multiphysics, including transport of diluted species and electric current physics. The simulation conditions of the seven groups were the same as those of the OVA in vitro diffusion experiment. Ion transport was governed by the Nernst–Planck flux equation^51^. The diffusion rate, total amount diffused, and concentration distribution of OVA in the different simulation groups were calculated.

### Immunization studies

The in vivo immunization performance of the iontophoresis-driven MN patch was investigated. Female BALB/c mice (18 ± 2 g, 6-week old) were vaccinated and divided into six groups: control group, intradermal injection group, intramuscular injection group, MN group, 0.5 ITP group, and MN/0.5 ITP group (*n* = 5 per group). The hair on the right abdomen of each mouse was shaved 24 h before vaccination. The dried hydrogels (40 mg) were immersed in 250 µL of OVA solution (1 mg/mL) for 48 h at 4 °C to load OVA into the hydrogel for transdermal vaccination. The control, MN, 0.5 ITP and MN/0.5 ITP groups were vaccinated with iontophoresis-driven MN patches for 30 min. The control group was treated using iontophoresis-driven MN patches without skin penetration and iontophoresis. This procedure was repeated again after 2 weeks for the booster dose. Two weeks after the booster dose, blood from the retro-orbital plexus was collected, and serum was separated for antibody concentration analysis. OVA-specific IgG, IgG1, and IgG2a levels in the serum samples were measured using ELISA kits (MEIMIAN Industrial Co., Ltd., Jiangsu, China).

### Biosafety assessment

The abdominal skin recovery in the MN group and MN/0.5 ITP group after vaccination were observed. In MN/0.5 ITP group, blood from the retro-orbital plexus was collected before vaccination and 24 h after vaccination with the iontophoresis-driven MN patch. ALT and AST levels in the serum samples were measured using ELISA kits (Changchun Huili Biotech Co., Ltd., Jilin, China). Moreover, the skin, heart, liver, spleen, lungs and kidneys of the mice in the control group and MN/0.5 ITP group were harvested and stained with hematoxylin-eosin (H&E).

### Statistical analysis

The experimental data were calculated and are expressed as the mean ± standard deviation (SD). The differences between two groups were determined by Student’s *t* test. A one-sample *t* test was used to determine significant differences between the experimental and FEA values. *p* values < 0.05 indicated that the results were considered statistically significant (*p value < 0.05, **p value < 0.01, ***p value < 0.001).

## Supplementary information

Supplementary Information

Video S1

Video S2

## Data Availability

The data that support this study are available from the corresponding authors upon reasonable request.
