# Supplementary material for: Iontophoresis-driven microneedle patch for the active transdermal delivery of vaccine macromolecules
Source: Microsyst Nanoeng. 2023 Mar 27;9:35. doi: 10.1038/s41378-023-00515-1 (PMC10040928; doi:10.1038/s41378-023-00515-1)
Supplement: Supplementary file 1 — Supplementary Information [file 41378_2023_515_MOESM1_ESM.docx]

Supporting Information for

# Iontophoresis-Driven Microneedle Patch for the Active Transdermal Delivery of Vaccine Macromolecules

Ying Zheng ^a^, Rui Ye ^a^, Xia Gong ^a^, Jingbo Yang ^a^, Bin Liu ^a^, Yunsheng Xu ^b^, Gang Nie ^b^, Xi Xie ^c^, Lelun Jiang ^a,^ *

^a^ Guangdong Provincial Key Laboratory of Sensor Technology and Biomedical Instrument, School of Biomedical Engineering, Shenzhen Campus of Sun Yat-Sen University, Shenzhen, 518107, PR China;

^b^ Department of Dermatovenereology, The Seventh Affiliated Hospital, Sun Yat-sen University, Shenzhen, 518107, PR China;

^c^ State Key Laboratory of Optoelectronic Materials and Technologies, School of Electronics and Information Technology, Sun Yat-sen University, Guangzhou, 510006, PR China.

*Author for correspondence: jianglel@mail.sysu.edu.cn

1. **Design of the iontophoresis-driven device**

An iontophoresis-driven device was developed, as shown in Fig. S1. A double-layer printed circuit board (PCB) of iontophoresis-driven circuit with a size of 36.6×13.3 mm^2^ was fabricated from flexible PI. Rechargeable polymer lithium battery was selected as the power supply, whose capacity is 100 mAh.


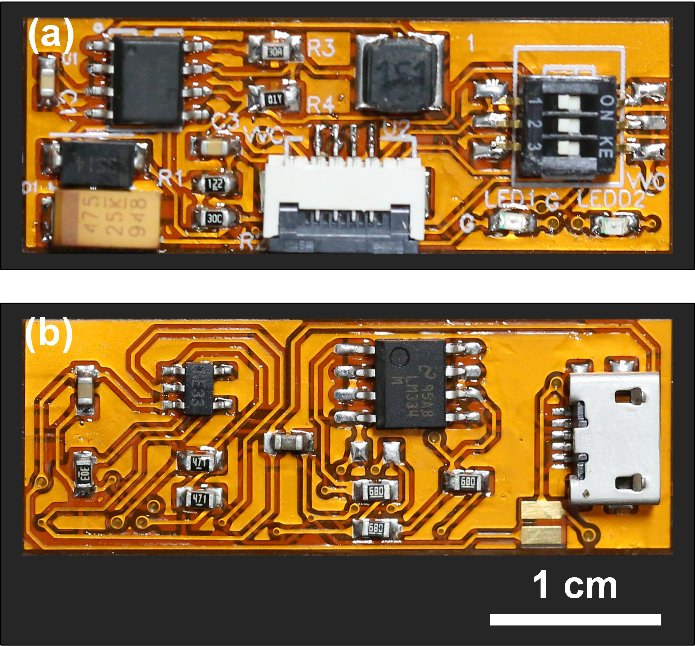


**Fig. S1** The PCB images of iontophoresis-driven device.

The schematic diagram of the iontophoresis-driven device circuit is shown in Fig. S2. The iontophoresis-driven circuit was mainly composed of the charging module, boosted module and constant current output module (Fig. S3-S7). The boosted module is equipped with MCP73831 chip, which is powered by a micro-USB port or a lithium battery (3.7 V), providing a continuous 3.0-3.7 V voltage. Patients also can use mobile power supply, smart phone and other devices to power the iontophoresis-driven device or charge the lithium battery through micro-USB port. The rechargeable lithium battery can be used directly as a power source for the iontophoresis-driven device to ensure the portability. The chip MC34063 can boost the voltage from 3.7 V to approximately 22 V. The boost equation is:

$V_{\text{out}}\text{ }=\text{ (1}+\frac{R_{7}}{R_{6}})\times\text{V}_{\text{ref}}$ (S1)

in which, *V*_out_ is the output voltage, *V*_ref_ = 1.25 V, *R*_6_ and *R*_7_ are the voltage divider resistances, as shown in Fig. S2. Since the skin resistance is very high, the lithium battery voltage (approximately 3.7 V) has to raise at approximately 20 V to output a stable constant current. According to the voltage boost formula of the chip MC34063 can boost the voltage from 3.7 V to 22.08 V when the resistances of *R*_6_ and *R*_7_ are selected as 1.2 kΩ and 20 kΩ, respectively. The core chip of the constant-current module is LM334, which can adjust the output currents (0.5 mA, 1 mA, 1.5 mA and 2 mA) by connecting different resistors.

**
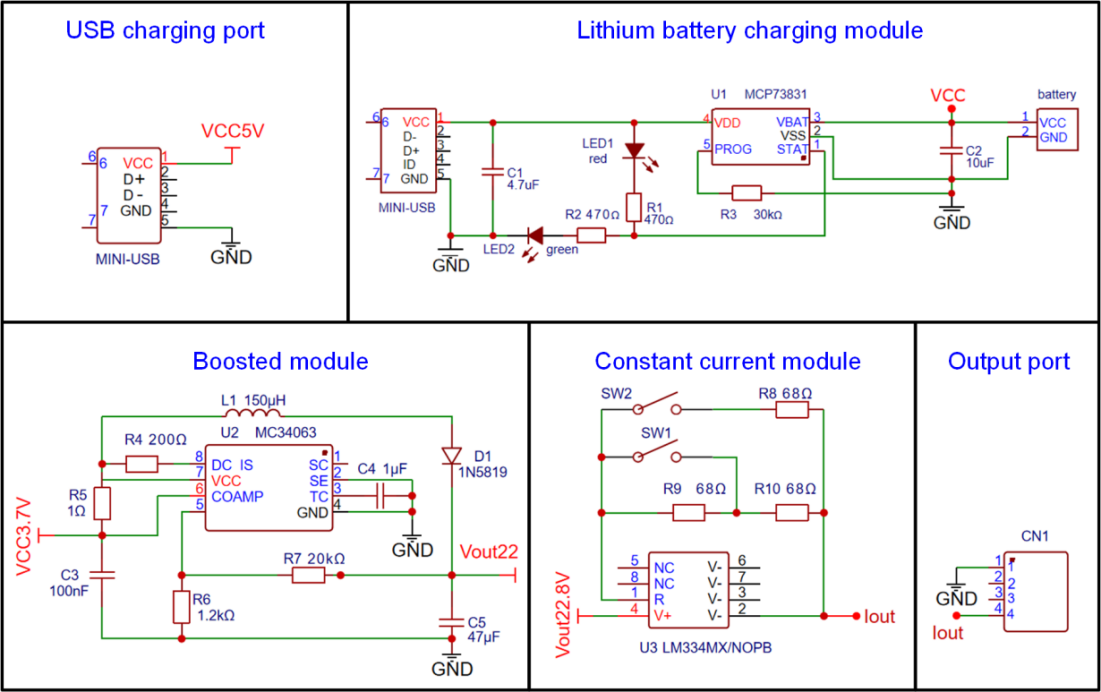
**

**Fig. S2** Schematic diagram of the iontophoresis-driven device.


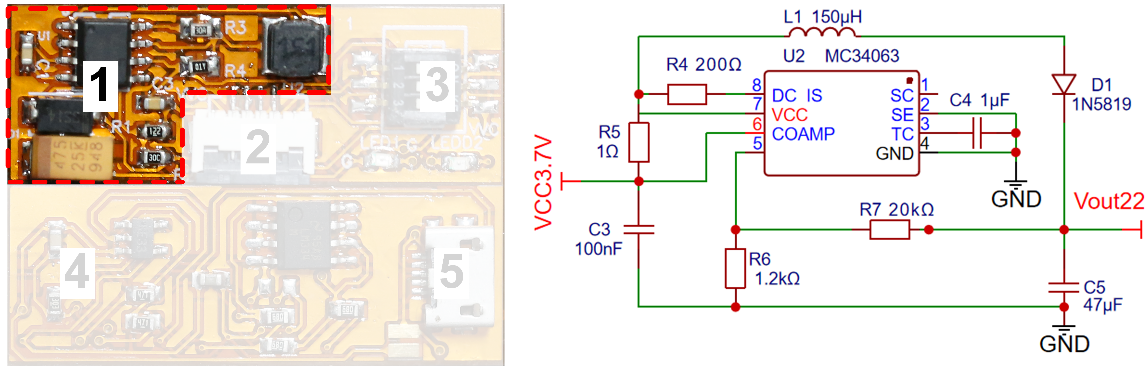


**Fig. S3** Schematic diagram of the boosted module.


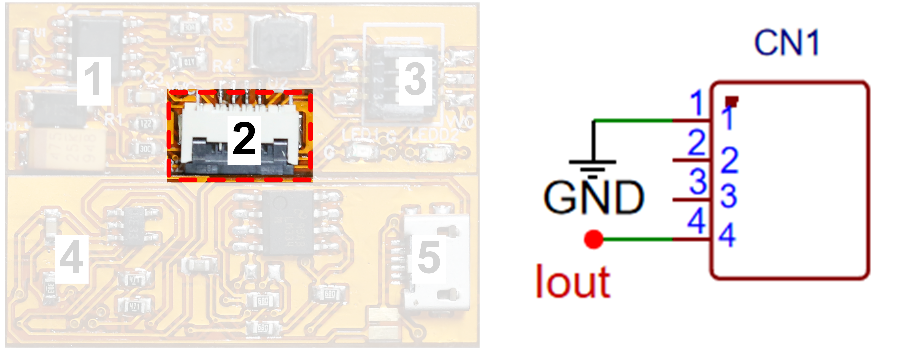


**Fig. S4** Schematic diagram of output port.


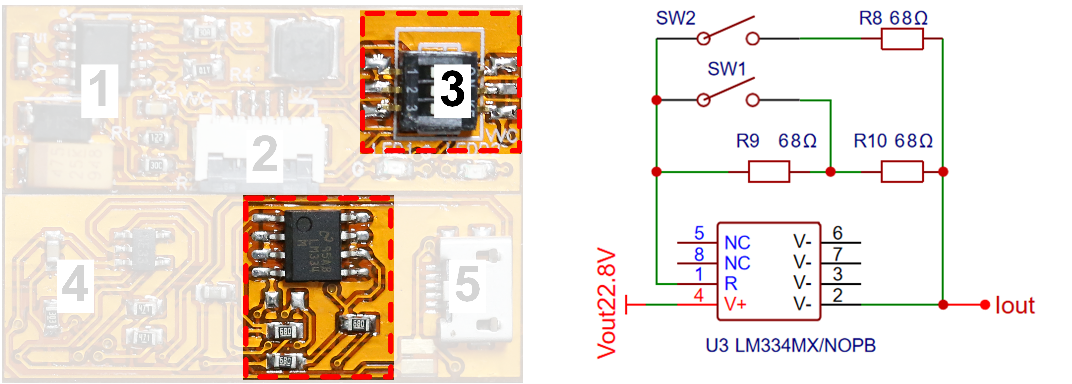


**Fig. S5** Schematic diagram of constant current module.


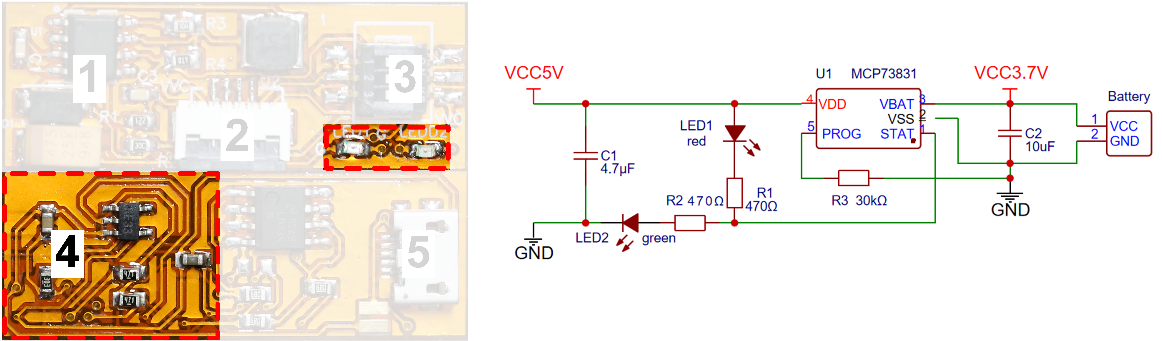


**Fig. S6** Schematic diagram of lithium battery charging module.


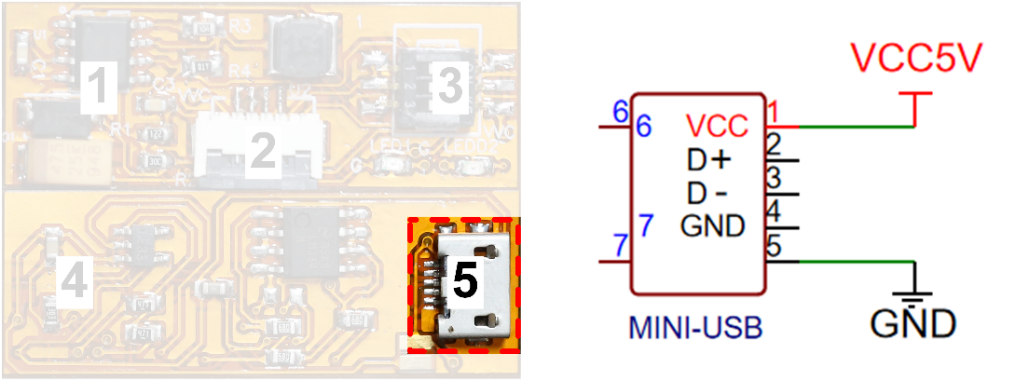


**Fig. S7** Schematic diagram for USB charging port.

**2.** **The charging and output performance of iontophoresis-driven device**

The charging and output performance of the iontophoresis-driven device was investigated. The individual skin resistance is different. The output voltage of iontophoresis-driven device is linearly proportional to the loading resistances ranging from 5 to 20 kΩ (typical skin resistance), as shown in Fig. S8a, indicating the iontophoresis-driven device can output a constant current under different resistances. The iontophoresis-driven device was connected to a power adapter (5 V, 0.5 A) to charge the fully discharged lithium battery, and the charging current is shown in Fig S8b. After charging of lithium battery for 80 min, the charging current dropped gradually, indicating the charging process had been finished. In order to test the power supply capability of the selected lithium battery, a 20 kΩ resistor was connected to the output port of iontophoresis-driven device. The discharging current of lithium battery, voltage of lithium battery, and loading voltage were recorded, as shown in Fig S8c-e. The lithium battery can output a constant current of 0.5 mA within 60 min. *In vitro* and *in vivo* transdermal vaccine delivery requires the iontophoresis-driven device to maintain the constant current output for 30 min. Therefore, the design of iontophoresis-driven device can satisfy the requirement of transdermal vaccine delivery using the iontophoresis-driven MN patch.

**
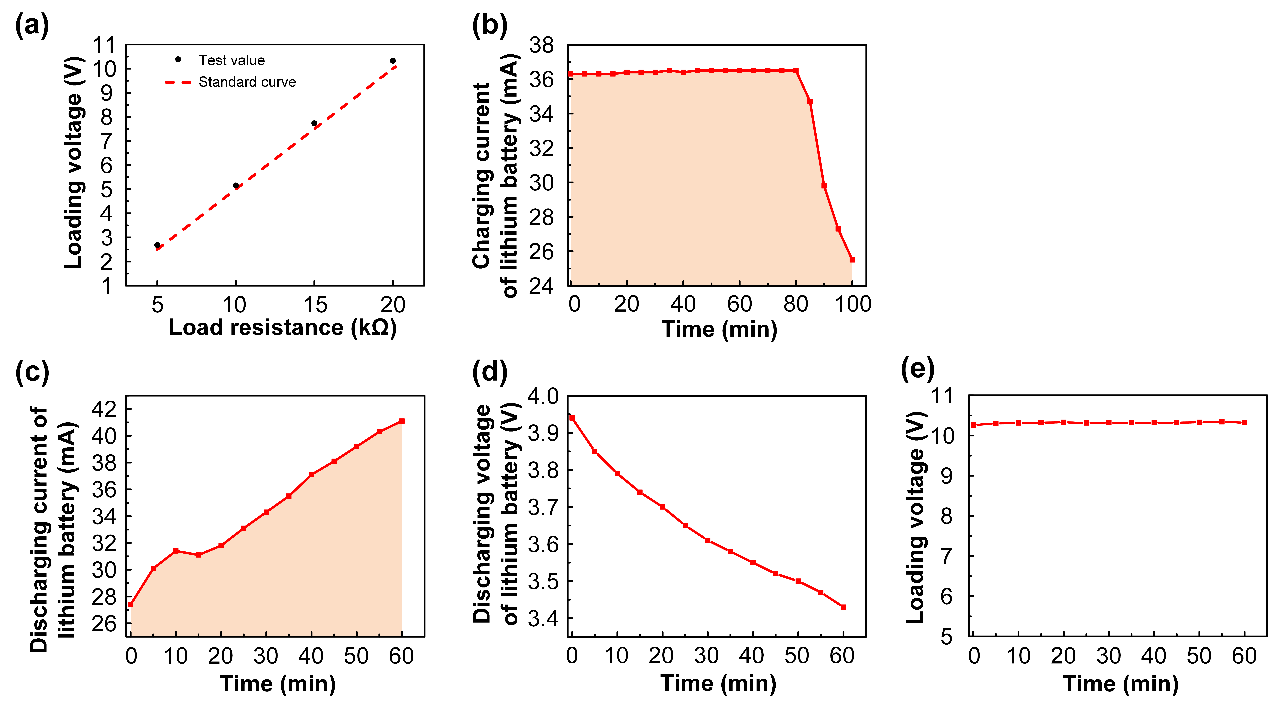
**

**Fig. S8** The performance tests of the iontophoresis-driven device. (a) The output loading voltage of iontophoresis-driven device under various loading resistors. (b) The charging current of lithium battery. (c) The discharging current of lithium battery, (d) the discharging voltage of lithium battery, and (e) the loading voltage of iontophoresis-driven device during discharging process.

**3. OVA-FITC distribution in hydrogel**

Confocal laser scanning microscopy (CLSM, FV3000, Olympus, Japan) was used to observe the drug distribution in the hydrogel, as shown in Fig. S9. OVA-FITC was evenly distributed in the hydrogel.

**
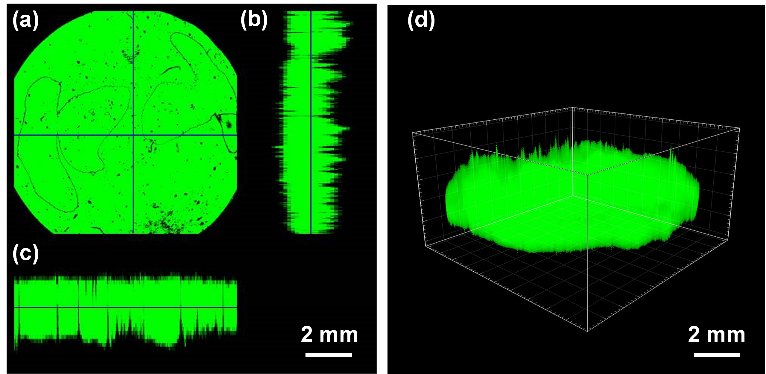
**

**Fig. S9** CLSM images of hydrogel loaded with OVA-FITC, exhibiting the distribution of OVA-FITC along the (a) Z, (b) Y, and (c) X-axis cross sections of the hydrogel. (d) 3D confocal reconstruction image of the OVA-FITC distribution in the hydrogel.

**4. Drug release performance of the hydrogel**

The hydrogels loaded with OVA were immersed in 15 mL PBS buffer to test its drug release performance. Three groups, including sponge group, hydrogel applied with 1 mA iontophoresis current group, and hydrogel group, were tested. Two carbon electrodes were loaded with 1 mA current, as shown in Fig. S10.


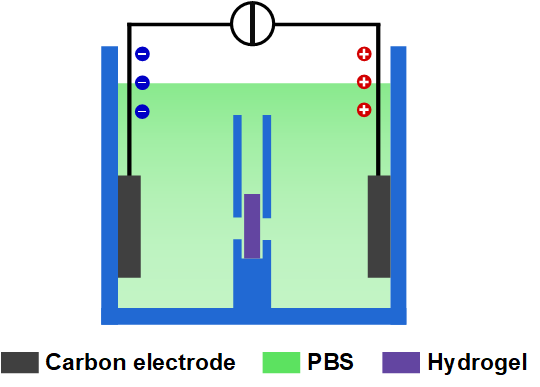


**Fig. S10** Schematic diagram of OVA release test of hydrogel loaded with 1 mA current.

**5. Hydrogel optical image during compression test**

The hydrogel was compressed using the universal material testing machine. The cylindrical hydrogel block was compressed at a speed of 0.1 mm/s. The hydrogel block was repeatedly compressed for 100 cycles at a limit strain of 70 %. As shown in Fig. S11, Optical images during the experiment showed that the shape of the hydrogel did not change and there was no damage after 100 times of repeated compressions.


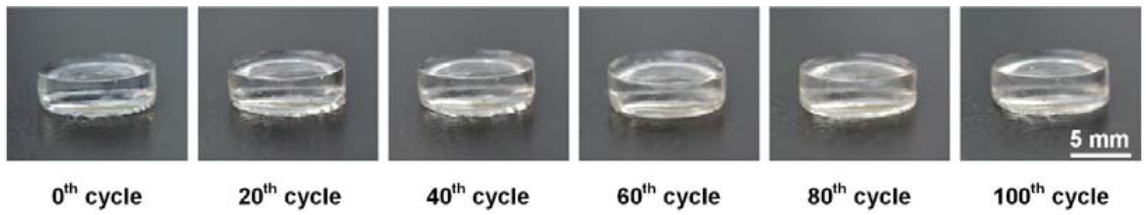


**Fig. S11** Hydrogel optical image during 100 times of repeated compressions.

**6. Linear fit of permeation amount**

The experimental cumulative permeation amount of OVA almost linearly increased with the iontophoresis-driven current, as shown in Fig. S12a. The simulated cumulative permeation amount of MN/ITP group also linearly increased with the iontophoresis-driven current, as shown in Fig. S12b. The experimental and simulated results are well consistent, demonstrating the delivery effectiveness and controllability of active iontophoresis.


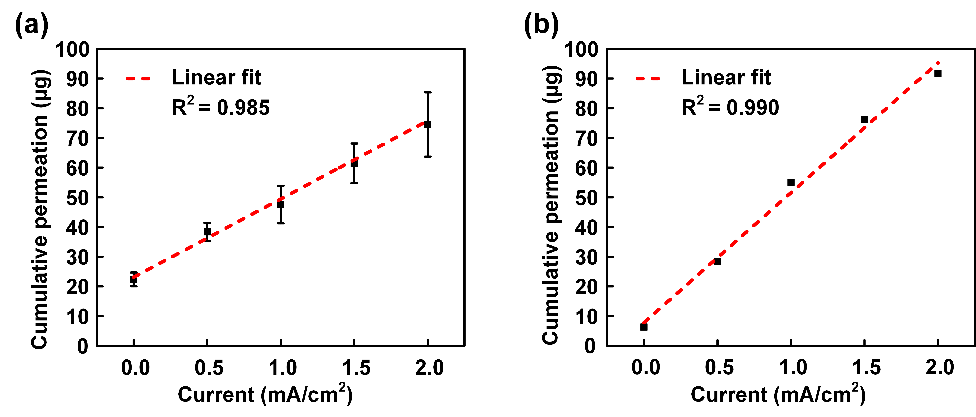


**Fig. S12** (a) The relationship between the experimental cumulative permeation amount and iontophoresis current. (b) The relationship between the simulated cumulative permeation amount and iontophoresis current.

**7.** **Numerical simulation of transdermal drug delivery process**

The transdermal vaccine delivery process of iontophoresis-driven MN patch combined skin penetration and iontophoresis was analyzed using finite element analysis (FEA) method. The FEA model of iontophoresis-driven MN patch was established using COMSOL Multiphysics, as shown in Fig. S13. The FEA models and parameters are listed in Table S1. The synergistic permeation effect of MN puncture and iontophoresis of the iontophoresis-driven MN patch was calculated using the potential coupling multiphysics including Transport of Diluted Species and Electric currents physics. The simulation conditions of seven groups were the same as those of OVA *in vitro* diffusion experiment. Ion transport was governed by the Nerst-Planck flux equation ^1^.

$J_{\text{i}}\text{ }=\text{ }uc_{\text{i}}\text{ - }D_{\text{i}}\nabla c_{\text{i}}\text{ - }\frac{z_{\text{i}}c_{\text{i}}D_{\text{i}}\text{F}\nabla\phi}{\text{RT}}$ (S2)

in which *J*_i_ is the transport flux of ion i, *u* is the fluid velocity, *φ* is the electrical potential, F is the Faraday constant, R is the gas constant, and T is the absolute temperature of the environment for ion i. Besides, *c*_i_, *D*_i_ and *z*_i_ in the equation represent the concentration, diffusion coefficient and charge of ion i, respectively. The three terms on the right of the equation represent the convection flux, diffusion flux and electromigration flux, respectively.

The simulation groups, including the control group, MN group, 1 ITP group, MN/0.5 ITP group, MN/1 ITP group, MN/1.5 ITP group and MN/2 ITP group were analyzed. The diffusion rate, total diffusion amount, and concentration distribution of OVA in different simulation groups were calculated.


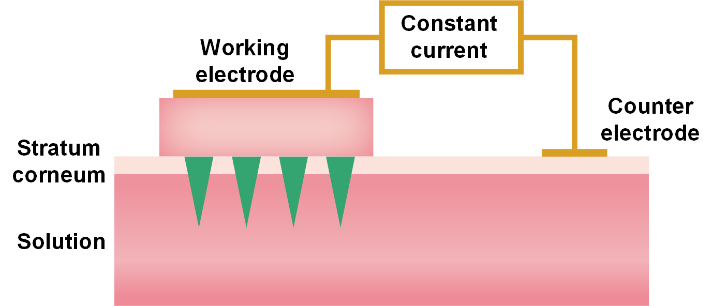


**Fig. S13** Schematic diagram of the simulation model of iontophoresis-driven MN patch.

**Table S1** The specific parameters of numerical simulation

| **Parameters** | **Value** | **Unit** | **Paraphrase** |
| --- | --- | --- | --- |
| Hydrogel radius | 6.2 | mm | Radius of hydrogel |
| Hydrogel thickness | 2 | mm | Thickness of hydrogel |
| SC thickness | 0.02 | mm | Thickness of SC |
| Conductivity-h | 0.15 | S/m | Conductivity of hydrogel |
| Conductivity-s | 1.5 | S/m | Conductivity of PBS solution ^2^ |
| Diffusion-h | 5e-11 | m^2^/s | Diffusion coefficient of hydrogel |
| Diffusion-s | 1e-10 | m^2^/s | Diffusion coefficient of PBS solution |
| Diffusion-sc | 0.00025 |  | Relative diffusion coefficient of SC to water ^3^ |
| Concentration-h | 0.0139 | mol/m^3^ | Initial concentration of OVA in hydrogel |
| Z_OVA_ | -17 |  | Net negative charge of OVA in PBS (pH= 7.4) |
| Q | 0, 5, 10, 15 and 20 | A/m^2^ | Iontophoresis-driven currents. |

**8. Biosafety tests of iontophoresis-driven MN patch**

The micro-pores in the skin of the mouse back poked by MN were healed within 40 min without erythema or lesions, as shown in Fig. S14. Furthermore, the skin sections of mice in control group and MN/0.5 ITP group were cut and stained with H&E, as shown in Fig. S15. There was no significant infiltration of inflammatory cells in the skin. The administration using iontophoresis-driven MN patch will not cause adverse reactions to the skin.


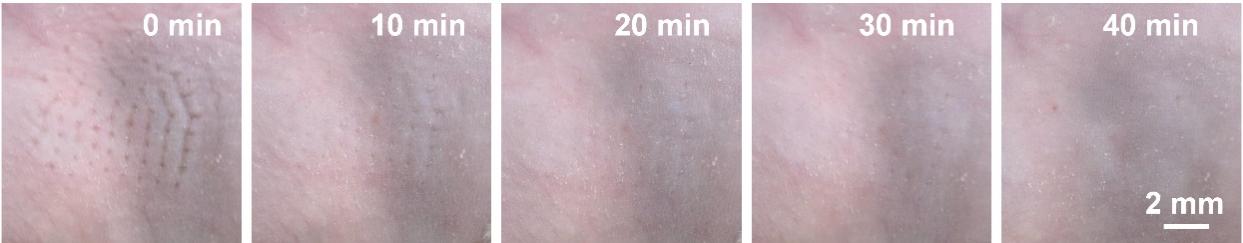


**Fig. S14** The skin recovery process of mouse after MN puncture


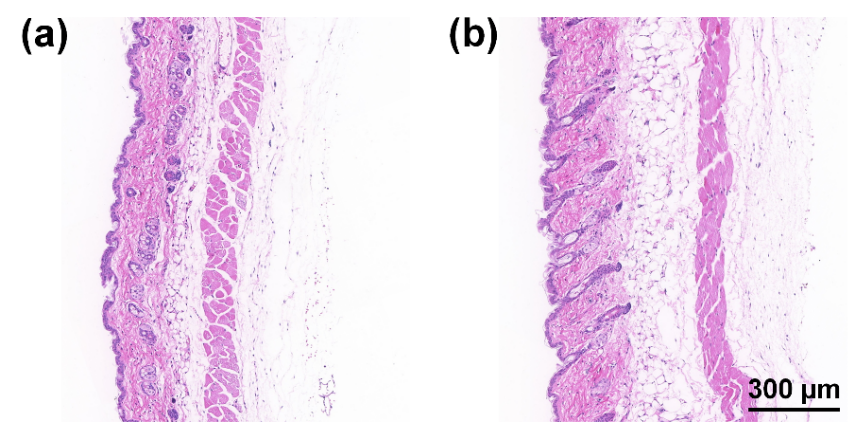


**Fig. S15** H&E stained of skin sections of (a) control group, and (b) MN/0.5 ITP group.

**9. Mechanical tests of the iontophoresis-driven MN patch**

The skin penetration performance of iontophoresis-driven MN patch was tested using the universal material testing machine (Instron, 5543A, Boston, USA), as shown in Fig. S16a. The rat skin was fixed on the polystyrene foam. Iontophoresis-driven MN patch was gradually pressed at 4 mm, then hold for 10 s, and subsequently released. The press and release speed was 0.1 mm/s. The resistance force and loading displacement were recorded. he fracture performance of MN was tested using the universal material testing machine (Instron, 5967, Boston, USA), as shown in Fig. S16b. MN was moved toward and pressed on a stainless steel plate till the resistance force reached at 3.5 kN at a speed of 0.1 mm/s. The resistance force and loading displacement were also recorded. The compressed MN was observed using optical microscopy (Keyence, VHX-5000, Osaka, Japan), as shown in Fig. S17.


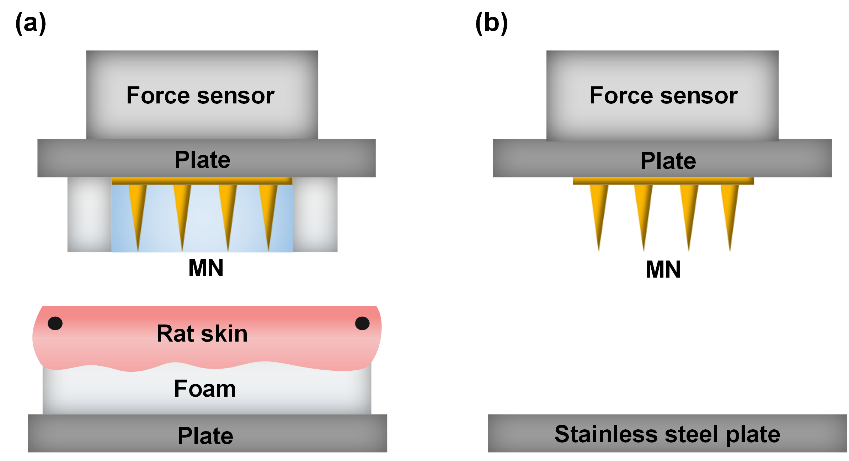


**Fig. S16** Schematic illustration of (a) skin penetration test, and (b) MN fracture test.

**
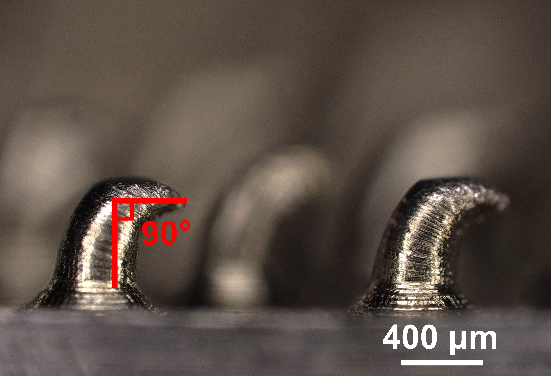
**

**Fig. S17** The image of bent microneedles after fracture test.

**10. *In vitro* OVA** **transdermal delivery test of iontophoresis-driven MN patch**

*In vitro* OVA delivery performance of iontophoresis-driven MN patch was tested using a self-designed Franz diffusion cells (TP-3A, Albert Tech., China), as shown in Fig. S18. A donor chamber and a receptor chamber were designed for the *in vitro* experiment. The receptor chamber (17 ml) was filled with a phosphate buffer solution (PBS, pH= 7.4). The rat skin with a size of 3×4 cm^2^ was fixed on the receptor chamber. Iontophoresis-driven MN patch was assembled on the donor chamber, as shown in Fig. S18. The self-developed iontophoresis-driven device was used for iontophoresis-driven delivery.


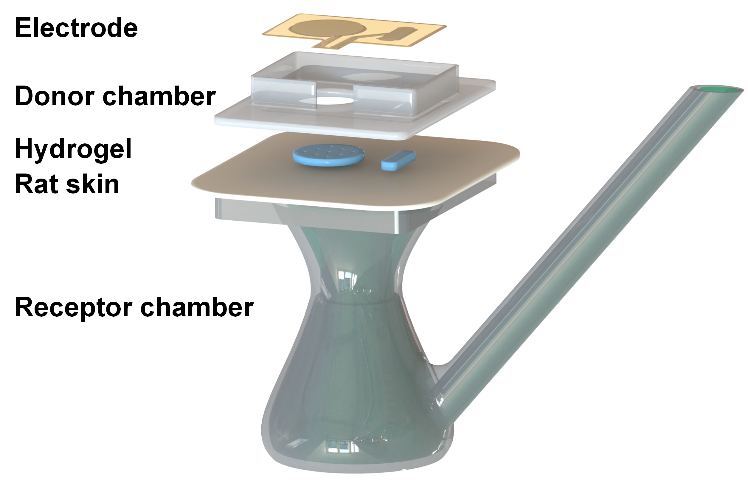


**Fig. S18** Illustration of self-developed vertical Franz diffusion cell.

*In vitro* OVA transdermal delivery tests were divided into 7 groups, as shown in Table 1. The schematic diagram of transdermal delivery tests of control, MN, ITP and MN/ITP groups is shown in Fig. S19. During the test, the receptor chamber was continuously stirred at an environment of 37 ℃. A sample of 100 µL was removed from the receptor chamber every 10 min and replaced with the same amount of PBS solution.


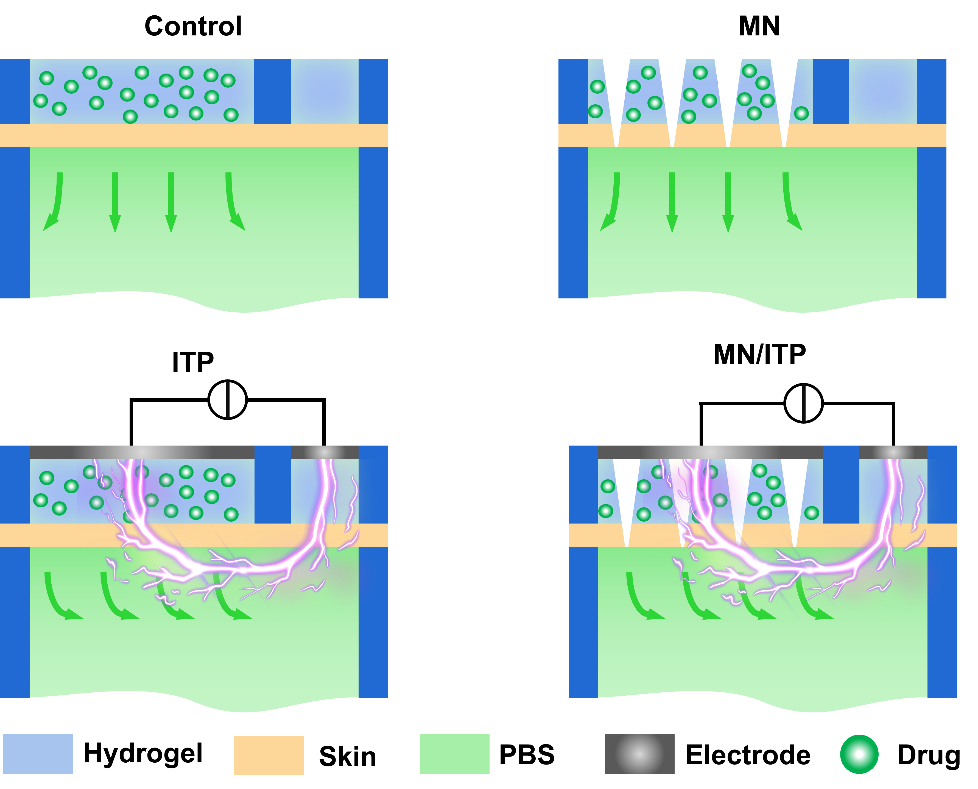


**Fig. S19** Schematic diagram of transdermal delivery tests of control, MN, ITP and MN/ITP groups.

**References:**

1 Wang, W., Chiang, T. Y., Velegol, D. & Mallouk, T. E. Understanding the efficiency of autonomous nano- and microscale motors. *J Am Chem Soc*. **135**, 10557-10565 (2013).

2 Kim, S. J., Ko, S. H., Kang, K. H. & Han, J. Direct seawater desalination by ion concentration polarization. *Nat Nanotechnol*. **5**, 297-301 (2010).

3 Li, X. *et al.* A Fully Integrated Closed-Loop System Based on Mesoporous Microneedles-Iontophoresis for Diabetes Treatment. *Adv Sci (Weinh)*. **8**, e2100827 (2021).
